# One Procedure, Multiple Nerves, Diverse Pain Phenotypes: A 4-Patient Case Series of Trigeminal Neuropathic Pain Following Facial Hyaluronic Acid Injections

**DOI:** 10.1093/asjof/ojag145

**Published:** 2026-07-09

**Authors:** Ortal Nachum, Shachar Zion Shemesh, Paz Kelmer, Itay Goor Aryeh, Lior Ungar

## Abstract

Facial hyaluronic acid (HA) injections are widely performed in aesthetic procedures, and their vascular complications are well recognized. In contrast, nonvascular neuropathic pain syndromes involving peripheral trigeminal nerve branches have received limited attention. In this study, the authors report a retrospective 4-patient case series of individuals evaluated at a tertiary headache and facial pain clinic for persistent facial pain or headache temporally associated with facial HA injections. Clinical assessment included injection history, symptom timing and evolution, anatomical pain distribution, focused neurologic examination, imaging when indicated, exclusion of alternative etiologies, and response to individualized treatment. The cases demonstrated heterogeneous peripheral trigeminal neuropathic phenotypes involving the infraorbital, infratrochlear, and supratrochlear nerve territories. Presentations included persistent trigeminal neuropathic pain, focal headache syndromes, and trigeminal neuralgia-like paroxysms. Two patients had infraorbital nerve involvement but distinct clinical manifestations, illustrating that injury or irritation of the same peripheral nerve may produce different pain phenotypes. All patients reported clinical improvement after individualized peripheral nerve-directed or local interventions, including hyaluronidase, targeted nerve blocks, pulsed radiofrequency, botulinum toxin, and neuropathic pain medications. These observations do not establish prevalence, causality, or treatment efficacy. However, they highlight the importance of careful procedural history, anatomical localization, exclusion of dental, otolaryngologic, central, and neurovascular causes, and early referral when symptoms persist. Prospective studies using standardized pain measures and targeted imaging are needed to define diagnostic criteria and management pathways.

**Level of Evidence: 4 (Therapeutic)**  
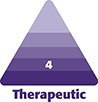

Facial hyaluronic acid (HA) fillers are widely utilized in contemporary aesthetic practice and are generally regarded as safe when administered by experienced practitioners. Their extensive application across multiple facial regions is driven by favorable aesthetic outcomes, reversibility, and a relatively low-reported complication rate. Nevertheless, a spectrum of adverse events has been described, ranging from mild, self-limited local reactions to severe, and potentially irreversible complications.^1,2^

The existing literature has largely focused on vascular complications associated with facial filler injections, including arterial embolization, skin necrosis, and visual loss.^3-6^ Although rare, these events typically present acutely, are well characterized, and have led to the development of established prevention and management strategies.^3-6^ As a result, vascular injury is a widely recognized risk and is routinely emphasized in injector training and preprocedural patient counseling.^1,4^

In contrast, nonvascular neurologic complications have received comparatively limited attention.^2,7^ Reviews addressing filler-related adverse events acknowledge the potential for peripheral nerve injury, compression, or neurapraxia; particularly in anatomically constrained facial regions but these complications are often mentioned only briefly and lack detailed clinical characterization.^2,7,8^ Peripheral branches of the trigeminal nerve course through narrow osteofibrous canals and confined soft-tissue planes adjacent to common injection sites, rendering them susceptible to mechanical compression, local inflammatory responses, or compromise of the vasa nervorum following filler placement.^8,9^

Neuropathic pain syndromes arising from peripheral trigeminal nerve involvement may manifest with heterogeneous clinical phenotypes, including persistent facial neuropathy, focal headache syndromes, or trigeminal neuralgia-like paroxysms.^7,10^ Symptom onset may be delayed, and the association with previous cosmetic injections may not be readily recognized by patients or clinicians. Consequently, these presentations are at risk of misdiagnosis as primary headache disorders or idiopathic trigeminal neuralgia, often leading to prolonged morbidity and extensive diagnostic evaluations.^2,7^

To date, the literature describing neuropathic pain following facial HA injections remains limited, consisting primarily of isolated case reports or brief discussions within broader reviews of filler-related complications.^10-12^ As a result, the clinical presentation, diagnostic pitfalls, and management considerations for these patients remain incompletely characterized. In this 4-patient case series, we describe distinct trigeminal neuropathic pain phenotypes temporally associated with facial HA injections, involving different peripheral trigeminal branches and variable responses to targeted treatment. The purpose of this report is not to estimate prevalence or establish causality, but rather to highlight hypothesis-generating clinical observations that may improve recognition, referral, and future prospective study design.

## METHODS

This retrospective 4-patient case series included consecutive patients evaluated between 2022 and 2025 at a tertiary, hospital-based pain clinic specializing in headache and facial pain disorders. Patients were identified through clinical records based on presentation with persistent head or facial pain temporally associated with facial HA injections. Because all patients were referred to a tertiary pain center after previous evaluation elsewhere, this cohort likely represents a selected group of more severe, persistent, or diagnostically challenging cases rather than the full spectrum of postinjection symptoms.

Data collection included detailed medical history, aesthetic procedural history, symptom onset and evolution, pain distribution, and neurologic examination findings. Particular attention was given to the anatomical correlation between pain distribution and peripheral trigeminal nerve branches. Neuroimaging was performed when clinically indicated to exclude central nervous system pathology or neurovascular conflict.

When available, details regarding the injection site, timing, injected material, and subsequent corrective procedures were reviewed. However, because several patients presented after a substantial delay from the original aesthetic procedure, precise information regarding HA product type, viscosity, injection depth, and injected volume was not consistently available. In addition, imaging or sonographic evaluation specifically demonstrating filler location relative to adjacent peripheral nerve structures was not performed systematically. These factors limited the ability to directly correlate filler deposition with nerve compression or irritation.

The clinical diagnosis of suspected peripheral trigeminal neuropathic pain or nerve entrapment was based on independent clinical criteria: (1) temporal association between symptom onset and facial HA injection, (2) pain distribution anatomically consistent with a specific peripheral trigeminal branch, (3) focal examination findings such as localized tenderness, sensory disturbance, or reproducible trigger zones when present, and (4) exclusion of alternative central, dental, otolaryngologic, or neurovascular etiologies. Response to peripheral nerve-directed interventions was not used as a defining diagnostic criterion; rather, it was recorded as supportive post hoc clinical evidence when present.

Management strategies were individualized and included pharmacologic therapy, hyaluronidase administration, targeted peripheral nerve blocks with or without corticosteroids, pulsed radiofrequency, and botulinum toxin injections.

Clinical outcomes were assessed descriptively based on patient-reported pain intensity, pain frequency, medication requirements, and functional impact during follow-up. Because of the retrospective design, standardized validated pain and functional instruments, such as numerical rating scales, headache disability scores, or quality-of-life questionnaires, were not collected systematically.

This study was approved by the IRB of the participating institution and was conducted in accordance with the Declaration of Helsinki. Written informed consent for inclusion and publication was obtained from all patients.

## RESULTS

### Patient Cohort and Overall Findings

Four patients presenting with persistent facial pain or headache following facial HA injections were included. All patients had undergone previous diagnostic evaluation elsewhere, including neurologic assessment and neuroimaging, without identification of central nervous system pathology or neurovascular conflict sufficient to explain the symptoms. Within this selected tertiary-care case series, clinical presentations were heterogeneous and involved different peripheral branches of the trigeminal nerve. Two patients demonstrated infraorbital nerve (V2) involvement with distinct clinical manifestations, whereas the remaining patients demonstrated clinical localization to the infratrochlear and supratrochlear nerves (V1). Patient characteristics, affected trigeminal branches, diagnostic considerations, therapeutic interventions, and descriptive outcomes are summarized in Table 1.

**Table 1.** Clinical Characteristics (Affected Trigeminal Branches and Management of Neuropathic Pain Following Facial HA Injections), Patient Characteristics, Pain Phenotypes, Diagnostic Considerations, Therapeutic Interventions, and Clinical Outcomes

Patient	Age/sex	Injection site	Affected trigeminal branch	Symptom onset	Pain phenotype	Initial diagnosis	Key interventions	Outcome
1	26/F	Lips, infraorbital region	Infraorbital nerve (V2)	Immediate	Persistent neuropathic pain with paresthesias	Dental pathology/facial pain	Hyaluronidase (injector), infraorbital steroid injections, amitriptyline	Partial improvement (∼50%); chronic neuropathy
2	27/M	Nasal dorsum (nonsurgical rhinoplasty)	Infratrochlear nerve (V1)	Delayed (weeks)	Persistent unilateral orbital headache	Worsening migraine	Infratrochlear nerve blocks, pulsed radiofrequency	Return to baseline headache frequency
3	29/F	Nasal radix (1 cc HA)	Supratrochlear nerve (V1)	Immediate	Chronic frontal headache	Primary headache disorder	Hyaluronidase (500 U), supratrochlear nerve blocks, botulinum toxin	Complete resolution with maintenance BoNT
4	54/F	Malar region, nasolabial fold	Infraorbital nerve (V2)	Early, progressive	Trigeminal neuralgia-like paroxysms	Trigeminal neuralgia	Carbamazepine, infraorbital foramen steroid injection	Sustained control on reduced carbamazepine dose

BoNT, botulinum toxin; HA, hyaluronic acid; V1, ophthalmic division of the trigeminal nerve; V2, maxillary division of the trigeminal nerve.

### Individual Patient Summaries

#### Patient 1

A 26-year-old woman with no medical history developed persistent facial pain following HA injections to the lips and infraorbital region. Symptoms began immediately after the procedure and consisted of burning pain with sensory disturbance localized to the left maxillary (V2) distribution. Because of the immediate onset of pain, hyaluronidase was administered by the injecting physician shortly after the procedure; however, symptoms failed to resolve and progressively worsened, extending to bilateral V2 territories.

The pain phenotype evolved into continuous neuropathic pain with superimposed paresthesias, exacerbated by speaking and tooth brushing. Dental pathology was initially suspected, and extraction of an impacted maxillary wisdom tooth relieved dental symptoms without improvement in facial pain. The clinical presentation was ultimately consistent with chronic infraorbital trigeminal neuropathy.

Targeted infraorbital nerve corticosteroid injections resulted in ∼30% pain reduction. Adjunctive treatment with amitriptyline (50 mg daily) provided additional benefit, with an overall symptom reduction of ∼50%. Residual neuropathic pain persisted despite multimodal management.

#### Patient 2

A 27-year-old man with a history of infrequent migraine attacks presented with a new pattern of persistent unilateral headache localized to the medial aspect of the right orbit. Symptoms developed several weeks after nonsurgical rhinoplasty performed with a dense HA filler. Transient postprocedural nasal bridge pain resolved within days, and no initial association was made between the procedure and the subsequent headache syndrome.

Before referral, extensive neurologic evaluation led to a diagnosis of worsening migraine. Preventive therapies, including beta-blockers and tricyclic antidepressants, provided limited benefit. Abortive treatment with multiple triptans was largely ineffective. Nonsteroidal anti-inflammatory drugs offered the most consistent relief but were limited by gastrointestinal adverse effects. Therapies targeting calcitonin gene–related peptide failed to improve symptoms.

At presentation to the pain clinic, focused examination revealed a highly localized trigger zone along the medial orbital region, consistent with infratrochlear nerve involvement. A diagnostic and therapeutic infratrochlear nerve block was followed by patient-reported reduction in pain intensity and frequency, with return toward his baseline headache burden. Repeated nerve blocks maintained symptom control, whereas pulsed radiofrequency provided only transient benefit.

#### Patient 3

A 29-year-old woman with no medical history developed bilateral frontal headaches shortly after injection of 1 mL of HA to the nasal radix. Symptoms began within 1 h and included ear pain and nasal congestion without evidence of infection. Over the following month, headaches became continuous and disabling.

Extensive otolaryngologic and neurologic evaluation was unrevealing. During the diagnostic workup, she was hospitalized with aseptic meningitis, which resolved with supportive care; however, headache symptoms persisted. Repeated brain MRI studies and lumbar punctures excluded central or inflammatory pathology, and multiple preventive and abortive therapies failed.

Following referral to the pain clinic, reassessment identified a clear temporal relationship between symptom onset and the nasal radix injection. Focal tenderness and pain reproduction along the supratrochlear nerve trajectory supported supratrochlear nerve entrapment neuropathy. Administration of 500 units of hyaluronidase to the nasal radix resulted in ∼50% symptom reduction. Subsequent supratrochlear nerve blocks provided transient benefit, whereas botulinum toxin injection into the glabellar complex was followed by marked and sustained patient-reported improvement, maintained with repeat injections every 3 months.

#### Patient 4

A 54-year-old healthy woman developed new-onset facial pain following HA injections to the malar region and nasolabial fold. Initial focal pain at the left nasal ala with radiation toward the upper lip evolved into frequent paroxysms of sharp, electric shock-like pain within the maxillary (V2) territory.

Neurologic evaluation resulted in a diagnosis of trigeminal neuralgia, and carbamazepine was initiated with good initial symptom control. Brain MRI demonstrated no neurovascular conflict. After ∼1 year, symptom exacerbation occurred, and escalation of carbamazepine failed to restore adequate control, prompting referral to a tertiary pain clinic.

Focused examination demonstrated reproducible pain localized to the infraorbital nerve territory. A diagnostic and therapeutic infraorbital foramen injection using corticosteroids was followed by substantial patient-reported pain relief and enabled tapering of carbamazepine to a maintenance dose of 400 mg daily, with sustained symptom control. Attempts at further dose reduction were associated with symptom recurrence.

## DISCUSSION

This 4-patient case series describes clinically heterogeneous trigeminal neuropathic pain phenotypes temporally associated with facial HA injections. Although facial fillers are widely perceived as low-risk procedures, their complication profile is well established, particularly in the context of central facial injections.^1,2^ Existing literature has focused predominantly on vascular complications, such as embolization, tissue ischemia, and visual loss, which are typically acute and readily recognized.^3-6^ In contrast, nonvascular neuropathic complications have received comparatively less clinical emphasis and may be overlooked in selected patients, particularly when symptoms are delayed, focal, or initially attributed to primary headache disorders or idiopathic trigeminal neuralgia.^2,7^ Given the small sample size and retrospective design, the present findings should be interpreted as hypothesis-generating rather than definitive evidence of a broader epidemiologic pattern.

### Peripheral Trigeminal Nerve Vulnerability in Facial Injections

Peripheral branches of the trigeminal nerve traverse narrow osteofibrous canals and confined soft-tissue planes in close proximity to common filler injection sites, including the nasal radix, infraorbital foramen, and nasolabial region.^8,9^ These anatomical characteristics predispose nerves to direct or indirect compression by filler material, postprocedural edema, local inflammatory response, or compromise of the vasa nervorum.^7-9^ Reviews of nonvascular filler complications have noted that product deposition or molding near foraminal exits may result in nerve compression or neurapraxia, most frequently involving the infraorbital nerve distribution.^2,7^ Unlike vascular events, which often present immediately, neuropathic sequelae may evolve gradually, contributing to delayed recognition and frequent misattribution of symptoms.

### Mechanistic Considerations: Entrapment vs Neuropathy

Entrapment neuropathy represents a pathophysiologic mechanism rather than a discrete diagnosis and may give rise to diverse trigeminal neuropathic presentations. Mechanical compression by filler material, inflammatory edema, or secondary fibrosis may lead to altered nerve conduction, ectopic firing, and peripheral sensitization.^7,10^ Expert consensus statements emphasize that nonvascular neurologic manifestations following filler injections are heterogeneous and frequently overlooked, particularly when symptom onset is delayed.^2,7^ The clinical improvement observed after peripheral nerve-directed or local interventions, including hyaluronidase administration, targeted nerve blocks, pulsed radiofrequency, and botulinum toxin, is consistent with a peripheral contribution in selected cases. However, because treatment response was assessed retrospectively and without standardized outcome measures, it should be interpreted as supportive rather than confirmatory evidence.

### Potential Role of Ultrasound

High-resolution ultrasound may have an important adjunctive role when the clinical history and pain distribution suggest a focal filler-related mechanism. In selected cases, ultrasound may help identify persistent filler deposits, local tissue changes, or anatomical relationships between filler material and superficial neurovascular structures. It may also guide targeted interventions, including hyaluronidase-assisted filler dissolution, hydrodissection, or peripheral nerve blocks. Importantly, ultrasound was not applied systematically in the present series, and therefore, its diagnostic yield cannot be assessed from these cases. Future prospective studies incorporating standardized ultrasonographic assessment may clarify whether ultrasound can improve anatomical confirmation, treatment selection, and procedural precision in patients with suspected filler-related trigeminal neuropathic pain.

### Integration With Existing Literature

Published reports of neuropathic pain following facial filler injections have largely been limited to isolated case descriptions. Benjamin et al reported persistent headache following nonsurgical rhinoplasty attributed to peripheral nerve entrapment, with rapid symptom resolution after hyaluronidase injection.^10^ Wei et al described a trigeminal neuralgia-like syndrome following HA and botulinum toxin injections, implicating secondary nerve compression as a plausible mechanism.^11^ Beyond HA fillers, Mingazova et al reported acute isolated trigeminal neuropathy following calcium hydroxylapatite injection, suggesting that diverse filler materials may contribute to peripheral nerve injury.^12^ Additional reports have documented infraorbital nerve injury presenting with sensory disturbance or anesthesia after soft-tissue filler injections, even in the absence of overt pain syndromes.^13^ Although these reports provide important mechanistic insights, they remain limited to single cases and do not fully capture the clinical breadth of these complications.

### Phenotypic Heterogeneity and Diagnostic Pitfalls

In contrast to previously published reports, the present series demonstrates multiple patients presenting with neuropathic pain syndromes involving different trigeminal branches and distinct clinical phenotypes following HA injection. Notably, 2 patients exhibited infraorbital nerve involvement with markedly different manifestations, ranging from persistent trigeminal neuropathy to paroxysmal trigeminal neuralgia-like pain. This observation underscores the heterogeneity of neuropathic pain phenotypes that may arise from injury to the same peripheral nerve and supports the concept of a neuropathic pain spectrum rather than discrete diagnostic entities.

A consistent feature across our cases, as well as those previously reported in the literature, was initial misdiagnosis as primary headache disorders or idiopathic trigeminal neuralgia. Comprehensive neurologic evaluation, including neuroimaging, failed to identify central or vascular pathology in all patients. In several cases, the association with previous cosmetic injections was not immediately disclosed by patients or initially considered clinically relevant by treating physicians, highlighting a diagnostic blind spot previously noted in reviews of filler-related complications.^1,2^

### Interpretive Constraints and Clinical Significance

The present series should be interpreted cautiously. The identification of 4 cases within a tertiary, hospital-based pain service does not allow estimation of incidence, prevalence, or relative risk after facial HA injection. Moreover, referral to a specialized pain clinic introduces selection bias toward persistent, severe, atypical, or treatment-refractory presentations. Nevertheless, the repeated combination of temporal association, focal anatomical localization, exclusion of alternative etiologies, and improvement after targeted peripheral interventions suggests that postinjection peripheral trigeminal neuropathic pain is a clinically relevant phenomenon worthy of further study. These observations are best viewed as hypothesis generating and may help clinicians consider this diagnosis in selected patients with persistent facial pain or headache after aesthetic procedures.

### Treatment Selection and Provisional Clinical Approach

Management should be individualized according to symptom duration, suspected mechanism, affected nerve branch, pain phenotype, previous treatment exposure, and timing of referral. In patients presenting shortly after injection, or when clinical or imaging findings suggest persistent filler-related compression, hyaluronidase may be considered. In patients with focal pain localization, reproducible tenderness, sensory disturbance, or trigger zones along a peripheral trigeminal branch, targeted peripheral nerve blocks may serve both diagnostic and therapeutic purposes. Neuropathic pain medications may be appropriate for continuous, bilateral, or incompletely responsive symptoms, whereas pulsed radiofrequency, botulinum toxin, cryotherapy, or other advanced interventions should be reserved for selected refractory cases and performed by clinicians experienced in facial pain management.

Potential assessment and treatment options for suspected trigeminal neuropathic pain following facial HA filler injection are presented in Table 2. Because the current evidence base is limited and heterogeneous, this framework should not be interpreted as a validated treatment algorithm or as a formal evidence-grading system. Rather, it provides a practical summary of assessment and treatment options that may be considered in selected patients. The evidence descriptors in Table 2 reflect the authors’ narrative synthesis of available case reports, expert reviews, mechanistic considerations, and the present case series and were not derived from a systematic review, meta-analysis, grading of recommendations assessment, development and evaluation (GRADE) assessment, or any other validated evidence-rating instrument (Table 2).

**Table 2.** Potential Assessment and Treatment Options for Suspected Trigeminal Neuropathic Pain Following Facial Hyaluronic Acid Filler Injection

Step	Intervention	Details	Evidence context (narrative; not formally graded)
1	Clinical assessment	Comprehensive evaluation of pain distribution, triggers, and associated symptoms. Identifies patterns suggesting nerve involvement.	Expert consensus; observational evidence
2	Ultrasound imaging	High-resolution ultrasound can detect filler deposition, nerve compression, and anatomical abnormalities. It also functions as a guide for interventions.	Emerging evidence (case reports/studies)
3	Initial interventions	*Hyaluronidase*: Used to dissolve filler when imaging or clinical findings suggest significant filler deposition near nerve pathways. *Hydrodissection:* Recommended in cases of direct nerve compression by surrounding tissue or filler, using carefully guided injections of saline or anesthetic solution to separate the nerve from surrounding structures. Ultrasound-guided imaging enhances precision, enabling detailed visualization of nerves, nearby vasculature, and fascial planes to optimize treatment outcomes.	Moderate evidence (multiple case reports and emerging ultrasound applications)
4	Pharmacological therapy	Neuropathic pain and chronic headache management:	High evidence (validated for chronic pain conditions)
	a. Tricyclic antidepressants	*Amitriptyline (Elatrolet)*: Starting dose 10-25 mg nightly; titrate gradually to 50-75 mg daily. *Nortriptyline*: Starting dose 10-20 mg nightly; titrate to a maximum of 75 mg daily. Effective for chronic neuropathic pain.	High evidence (validated in neuropathic pain studies)
	b. Anticonvulsants/Neuromodulators	*Gabapentin*: Starting dose 300 mg daily; gradually titrate up to 900-1800 mg daily depending on response. –*Pregabalin*: Starting dose 75 mg twice daily; can be titrated to 150-300 mg twice daily. Effective in dampening hyperactivity in pain pathways.	High evidence (well documented for neuropathic pain)
	c. SNRI (Serotonin-norepinephrine reuptake inhibitors)	*Duloxetine*: Starting dose 30 mg daily, titrate to 60 mg daily for neuropathic pain. –*Venlafaxine*: Starting dose 37.5 mg daily, titrate up to 75-225 mg daily depending on tolerance and effectiveness.	Moderate-to-high evidence
	d. Sodium channel blockers	*Carbamazepine*: Starting dose 100-200 mg twice daily; titrate gradually to 600-1200 mg daily. Standard for trigeminal neuralgia. *Lamotrigine (Lamictal)*: Starting dose 25 mg daily; titrate upwards weekly to a target dose of 100-200 mg daily.	High evidence (especially for trigeminal neuralgia)
	e. CGRP inhibitors	*Erenumab, Fremanezumab, Galcanezumab*: Preventive treatment tailored for chronic migraine or headache patterns that may involve trigeminal pathways. Dosing for erenumab: typically 70-140 mg subcutaneously monthly, depending on patient response.	High evidence (well documented for chronic migraine**)
	f. Nonsteroidal anti-inflammatory drugs	*Indomethacin*: Consider for paroxysmal hemicrania or other indomethacin-responsive headache syndromes. Dosing: typically initiated at 25 mg 2-3 times daily, and titrated based on response and tolerability.	High evidence (specific headache syndromes)
5	Targeted nerve blocks	Corticosteroid or anesthetic injections reduce inflammation and pain in specific nerve territories and can also serve as a diagnostic tool to confirm nerve-related pain.	Moderate evidence (case series)
6	Advanced interventions	*PRF*: Applied to affected nerves for short-term relief in cases where nerve pain persists despite other treatments. *Botulinum toxin:* Targeting affected muscle groups for sustained symptom reduction when other modalities fail. *Cryotherapy*: Superficial or targeted nerve cryoablation may offer prolonged relief of neuropathic pain symptoms by disrupting nerve signaling temporarily in localized areas.	Limited evidence (experimental and anecdotal)
7	Multidisciplinary approach	Referral to pain specialists for resistant cases. Psychological and rehabilitative support to address functional limitations and emotional distress.	Expert opinion (observational studies)

This table summarizes potential diagnostic and therapeutic options that may be considered according to clinical presentation. It is not intended as a validated treatment algorithm. Drug selection, dose titration, contraindications, and monitoring should follow local prescribing standards and patient-specific considerations. Evidence statements refer primarily to the broader literature on neuropathic pain, trigeminal neuralgia, chronic migraine, and facial pain, rather than to filler-related trigeminal neuropathic pain specifically.

Double asterisks (**) denote the authors’ narrative assessment that the available support is limited, consisting primarily of isolated case reports, small case descriptions, mechanistic rationale, or extrapolation from related trigeminal neuropathic pain conditions. This notation should not be interpreted as a formal level of evidence. No systematic review, meta-analysis, GRADE assessment, or validated evidence-rating method was performed.

CGRP, calcitonin gene–related peptide; HA, hyaluronic acid; PRF, pulsed radiofrequency.

### Clinical Implications for Plastic Surgeons and Aesthetic Practitioners

Recognition of peripheral trigeminal neuropathic pain as a possible, although apparently uncommon, complication after facial HA injection has practical implications for aesthetic practice. Persistent or delayed-onset facial pain or headache after filler injection, particularly when localized to a specific trigeminal branch or associated with focal tenderness, sensory disturbance, or reproducible trigger zones, should prompt consideration of a peripheral nerve mechanism. Early recognition may reduce prolonged diagnostic uncertainty and facilitate appropriate referral to neurology, pain medicine, or relevant surgical specialists. At the same time, clinicians should avoid assuming causality solely on the basis of previous filler exposure, and alternative dental, otolaryngologic, central, and neurovascular causes should be excluded. Future prospective studies incorporating standardized pain scales, functional measures, injection-material documentation, and targeted imaging are required to define optimal diagnostic and treatment pathways.

### Limitations

This study has several important limitations. First, the sample size is very small, and the manuscript should therefore be interpreted as a 4-patient case series rather than a study capable of defining prevalence, risk factors, or treatment efficacy. Second, the retrospective design limited the availability of standardized pain intensity, headache disability, functional outcome, and quality-of-life measures. Outcomes were based on clinical documentation and patient-reported improvement rather than prospectively collected validated instruments. Third, treatment strategies were heterogeneous and were influenced by symptom duration, previous therapies, referral timing, affected nerve branch, and clinician judgment; therefore, no firm comparative conclusions can be drawn regarding the relative effectiveness of hyaluronidase, nerve blocks, pulsed radiofrequency, botulinum toxin, or pharmacologic therapy.

Fourth, because patients were evaluated in a tertiary pain clinic, the cohort is subject to referral and selection bias and likely overrepresents more severe, persistent, atypical, or diagnostically challenging presentations. Fifth, precise details regarding HA product type, viscosity, injected volume, injection depth, and anatomical filler distribution were not consistently available, particularly in patients who presented long after the original aesthetic procedure. Dedicated ultrasound or imaging demonstrating filler location relative to peripheral nerve structures was not performed systematically, limiting mechanistic inference regarding direct compression, local inflammation, or entrapment. Finally, although temporal association, anatomical localization, exclusion of alternative etiologies, and response to targeted interventions support a plausible peripheral mechanism, causality cannot be established from this retrospective case series. These limitations underscore the need for prospective studies using structured clinical criteria, standardized outcome measures, and imaging protocols to develop evidence-based diagnostic and management algorithms.

## CONCLUSIONS

This retrospective 4-patient case series describes heterogeneous trigeminal neuropathic pain phenotypes temporally associated with facial HA injections. The cases highlight the importance of careful procedural history, anatomical pain localization, focused neurologic examination, and exclusion of alternative etiologies in patients presenting with persistent facial pain or headache after aesthetic procedures. Although improvement after peripheral nerve-directed interventions may support a peripheral mechanism, these observations remain hypothesis generating and do not establish prevalence, causality, or treatment efficacy. Prospective studies incorporating standardized pain and functional measures, detailed injection-material documentation, and targeted imaging are needed to validate diagnostic criteria and develop structured management pathways.
